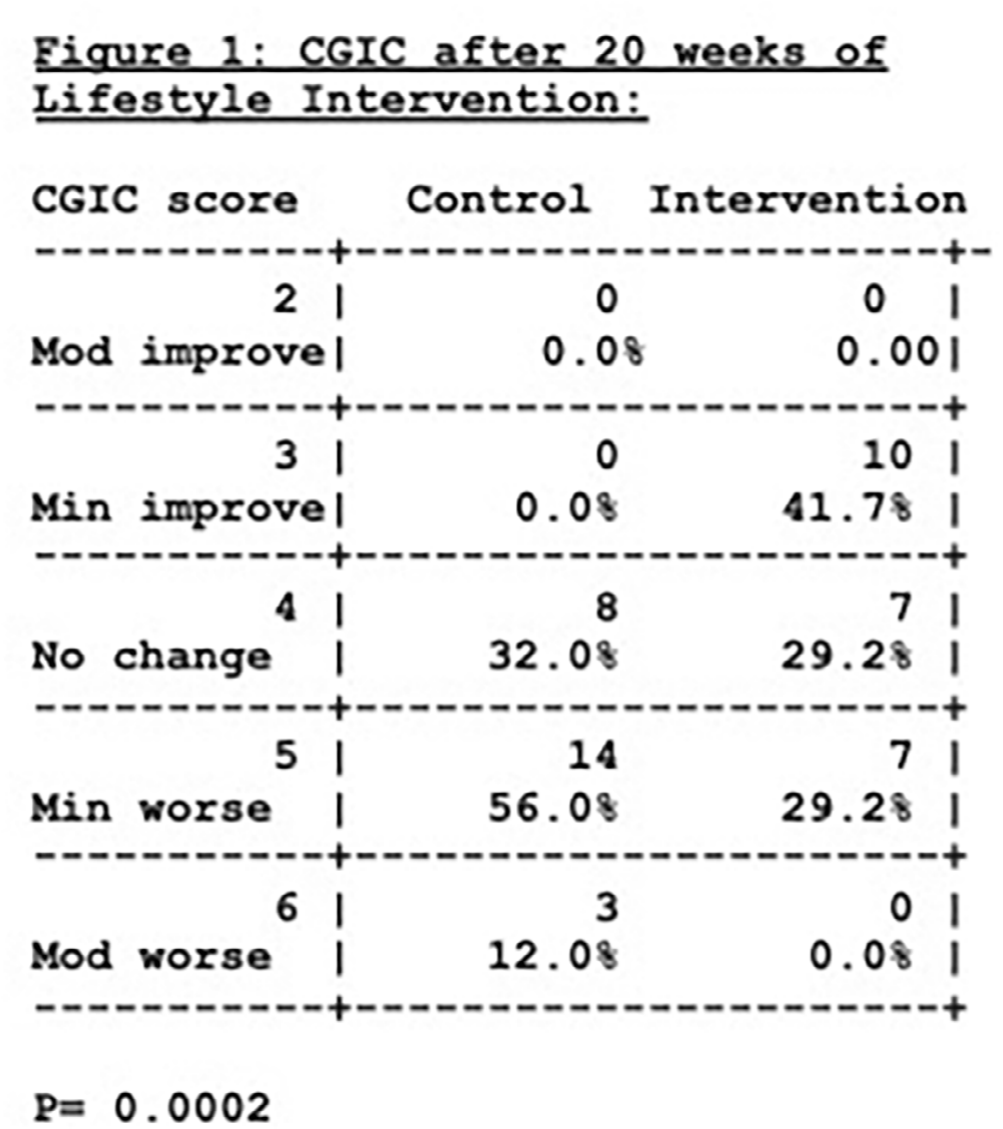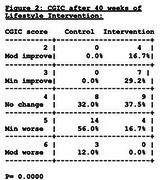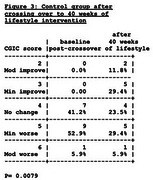# Effects of intensive lifestyle changes on the progression of mild cognitive impairment or early dementia due to Alzheimer's disease: Extended 40‐week results

**DOI:** 10.1002/alz70858_108967

**Published:** 2025-12-25

**Authors:** Dean Ornish, Catherine Madison, Miia Kivipelto, Colleen Kemp, Charles McCulloch, Douglas R. Galasko, Jon Artz, Jue Lin, Kim Norman, Anne Ornish, Sarah Tranter, Nancy DeLamarter, Noel Wingers, Carra Richling, Rima F. Kaddurah‐Daouk, Robert Knight, Daniel McDonald, Lucas Patel, Eric Verdin, Rudolph E. Tanzi, Steven E Arnold

**Affiliations:** ^1^ University of California, San Francisco, San Francisco, CA, USA; ^2^ CPMC, San Francisco, CA, USA; ^3^ Department of Neurobiology, Care Sciences and Society, Division of Clinical Geriatrics, Center for Alzheimer Research, Karolinska Institutet, Stockholm, Sweden; ^4^ Preventive Medicine Research Institute, Sausalito, CA, USA; ^5^ University of California, San Diego, La Jolla, CA, USA; ^6^ Renown Medical Group‐ Neurology, Reno, NV, USA; ^7^ Duke Institute for Brain Sciences, Duke University, Durham, NC, USA; ^8^ Department of Medicine, Duke University, Durham, NC, USA; ^9^ Department of Pediatrics, UC San Diego, La Jolla, CA, USA; ^10^ Buck Institute for Research on Aging, Novato, CA, USA; ^11^ Massachusetts General Hospital, Charlestown, MA, USA; ^12^ Massachusetts General Hospital, Harvard Medical School, Boston, MA, USA

## Abstract

**Background:**

We report the 40‐week follow‐up of a RCT to examine whether comprehensive lifestyle changes affect the progression of MCI or early dementia due to AD. Earlier results after 20 weeks showed significant improvement in cognition and function. [Ref: *Alzheimers Res Ther*. 2024 Jun 7;16(1):122. doi: 10.1186/s13195‐024‐01482‐z. PMID: 38849944; PMCID: PMC11157928.]

**Methods:**

A 1:1 multicenter randomized controlled phase 2 trial, ages 45‐90 with MCI or early dementia due to AD and MoCA score of 18 or higher. After the first 20 weeks, the intervention group continued to receive the lifestyle intervention for a total of 40 weeks. The intervention group was compared to the usual‐care randomized control group after 20 weeks since the control group crossed over and received the lifestyle intervention after 20 weeks.

**Results:**

Fifty‐one participants with MCI/AD enrolled, mean age 73.5. Active intervention and comparison groups did not differ in any baseline measures. After 40 weeks, there was significant improvement in the intervention group compared to the randomized nonintervention control group in the Clinical Global Impression of Change (CGIC) (*p* = 0.000, Figs 1 & 2), Clinical Dementia Rating–Sum of Boxes (*p* = 0.036), and Clinical Dementia Rating Global (*p* = 0.045). Improvement in Alzheimer's Disease Assessment Scale (ADAS‐Cog) after 20 weeks (*p* = 0.053) was not statistically significant after 40 weeks (*p* = 0.258). When comparing changes in the intervention group from 20 weeks to 40 weeks, there were significant additional improvements in the CGIC (*p* = 0.0000) and CDR‐SB (*p* = 0.0082) and no significant differences (i.e., changes were maintained) from 20 to 40 weeks in CDR Global (*p* = 0.1596) and ADAS‐Cog (*p* = 0.3892). After 20 weeks, the nonintervention control group crossed over and received the lifestyle intervention for 40 weeks. CGIC values are shown in Figure 3. The plasma Aβ42/40 ratio increased in the intervention group from baseline to 40 weeks when compared to the randomized control group (*p* = 0.0001). No significant between‐group changes in *p*‐tau 217 occurred.

**Conclusions:**

Comprehensive lifestyle changes may significantly improve cognition and function after 20 weeks in early dementia due to AD. After 40 weeks, these improvements were maintained in two measures and showed further improvement after 40 weeks in two measures.